# Sequence Analysis and Potentials of the Native *RbcS* Promoter in the Development of an Alternative Eukaryotic Expression System Using Green Microalga *Ankistrodesmus convolutus*

**DOI:** 10.3390/ijms13032676

**Published:** 2012-02-28

**Authors:** Tran Thanh, Vu Thi Quynh Chi, Hishamuddin Omar, Mohd Puad Abdullah, Suhaimi Napis

**Affiliations:** Bis Nam Ky Khoi Nghia, District 3, Ho Chi Minh City, Vietnam; 1 Department of Cell and Molecular Biology, Faculty of Biotechnology and Biomolecular Sciences, Universiti Putra Malaysia, 43400 UPM-Serdang, Selangor Darul Ehsan, Malaysia; E-Mails: tranthanhrriv@yahoo.com (T.T.); quynhchi_rriv@yahoo.com (V.T.Q.C.); aspuad@biotech.upm.edu.my (M.P.A.); 2 Rubber Research Institute of Vietnam, 236; 3 Department of Biology, Faculty of Science, Universiti Putra Malaysia, 43400 UPM-Serdang, Selangor Darul Ehsan, Malaysia; E-Mails: hishamom@science.upm.edu.my; hishamspirulina@gmail.com

**Keywords:** *Ankistrodesmus convolutes*, *gusA*, promoter, *RbcS*, gene expression, transcription start site

## Abstract

The availability of highly active homologous promoters is critical in the development of a transformation system and improvement of the transformation efficiency. To facilitate transformation of green microalga *Ankistrodesmus convolutus* which is considered as a potential candidate for many biotechnological applications, a highly-expressed native promoter sequence of ribulose-1,5-bisphosphate carboxylase/oxygenase small subunit (*AcRbcS*) has been used to drive the expression of β-glucuronidase (*gusA*) gene in this microalga. Besides the determination of the transcription start site by 5′-RACE, sequence analysis revealed that *AcRbcS* promoter contained consensus TATA-box and several putative *cis*-acting elements, including some representative light-regulatory elements (e.g., G-box, Sp1 motif and SORLIP2), which confer light responsiveness in plants, and several potential conserved motifs (e.g., CAGAC-motif, YCCYTGG-motifs and CACCACA-motif), which may be involved in light responsiveness of *RbcS* gene in green microalgae. Using *AcRbcS* promoter::gusA translational fusion, it was demonstrated that this promoter could function as a light-regulated promoter in transgenic *A. convolutus*, which suggested that the isolated *AcRbcS* promoter was a full and active promoter sequence that contained all *cis*-elements required for developmental and light-mediated control of gene expression, and this promoter can be used to drive the expression of heterologous genes in *A. convolutus*. This achievement therefore advances the development of *A. convolutus* as an alternative expression system for the production of recombinant proteins. This is the first report on development of gene manipulation system for unicellular green alga *A. convolutus*.

## 1. Introduction

As photoautotrophs, green microalgae have many advantages as expression systems for the production of recombinant proteins: (i) they can be cultured simply, rapidly and economically although they are eukaryotic; (ii) they can produce complicated eukaryotic proteins which need post-translational modification [1]; and (iii) they are considered as safe food because they are free from human pathogens and endotoxins [2]. Although a number of expression systems have been developed and are available for the production of recombinant proteins, it is necessary to develop alternative expression systems which offer distinct advantages in terms of protein yield, ease of manipulation, cost of operation and that are generally regarded as safe. The heterologous protein expression systems using green algae meet these criteria. On using algae as the expression systems, it is necessary to use the strong constitutive and native promoters or the promoters from very close species to overcome the hurdles of the low-efficient expression of the transgenes [3–6].

Among the popular strong constitutive promoters, cauliflower mosaic virus 35S (CaMV35S) was used to drive the expression of foreign genes in several green microalgae species such as *Chlamydomonas reinhardtii* [7], *Chlorella kessleri* [8], *Chlorella ellipsoidea* [9] and *Dunaliella salina* [10,11]; however, all attempts using this promoter to express foreign genes in *Chlamydomonas reinhardtii* have failed [12]. The other constitutive promoters, Simian virus 40 (SV40) and ubiquitin (*Ubi*) promoters, were used in transformation systems of *Chlamydomonas reinhardtii* [13], *Haematococcus pluvialis* [14] and *Chlorella ellipsoidea* [15], respectively. The low-efficiency or the failure to express the foreign genes using these heterologous promoters reported in these algae species may be due to the lack of necessary regulatory elements or the poor codon usage of the transgenes, and these problems can be overcome by using the native promoters and codon optimization in the transformation constructs [16,17].

In order to improve the efficiency transformation of green microalgae as well as to establish the transformation procedures, several native promoters have been isolated and used. As such, the native heat shock protein 70 (*HSP70*) promoter has allowed the efficient transgene expression in green microalga *Chlamydomonas reinhardtii* [4,17]. Moreover, the promoter of abundant protein of photosystem I complex (*PsaD*) genes have proved to be of high value for efficient expression of chimeric constructs [18]. Similarly, the native promoter of ribulose-1,5-bisphosphate carboxylase/oxygenase small subunit (*RbcS*) gene has given high transformation efficiency in transgenic *C. reinhardtii* [7,19–23] and *D. salina* [24]. Likewise, the *RbcS* promoter isolated from *D. tertiolecta* resulted in high expression level of the heterologous gene in transgenic *C. reinhardtii* [5]. Therefore, the highly active homologous promoter of *RbcS* genes is currently a priority choice to drive the expression of heterologous proteins in green microalgae.

The promising opportunity to use the fast growing green microalgae species as transgenic green cell factories has already resulted in several business start-ups during the last few years. As a fast growing alga producing appreciable amount of carotenoids and polyunsaturated fatty acids, especially lute [25], green microalga *A. convolutus* is being considered as an interesting candidate for many biotechnological applications. The simple, rapid and cheap culture of *A. convolutus* offers great advantages in bioengineering this species for producing valuable polypeptides and proteins safely and economically. Furthermore, the ability of *A. convolutus* to form floating aggregates during its normal growth besides other beneficiary attributes facilitates harvesting the biomass as well as the natural products and recombinant proteins [26]. Therefore, this species has high potential of being used as sources of lutein in poultry industry. Prospectively, *A. convolutus* is to be used as economic host to express the therapeutic proteins that can be served as an oral vaccine for poultry using the whole cell as the vector, as such no protein purification steps are needed meanwhile the animals are supplied with the amount of lutein in the cells. In order to establish an alternative expression system using *A. convolutus*, it is important to develop gene manipulation systems for this alga. Together with the knowledge of promoters and expression patterns of transgenes in other green microalgae species, exploiting the constitutive and native promoters is an important means for overcoming the hurdles of the low-efficient expression of transgenes in these algae. In the present study, the sequence of *RbcS* promoter from *A. convolutus* was analyzed and confirmed its ability to drive the expression of foreign genes in this alga. Also, the influence of light on the regulation of *AcRbcS* promoter activity was also reported. This is the first report on the development of gene manipulation system for green microalga *A. convolutus*.

## 2. Results and Discussion

### 2.1. Sequence Analysis of *AcRbcS* Promoter

**Figure 1:**
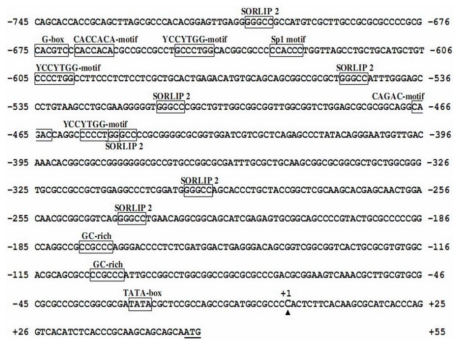
Nucleotide sequence of the *AcRbcS* promoter. The start codon (ATG) was underlined and the putative transcriptional start site (TSS) was indicated by a vertical arrow and referred to as position +1. The putative TATA-box and other *cis*-acting elements were boxed and the names are given above the elements. The accession number of the *AcRbcS* promoter deposited in the GenBank database is JF802127.

The *AcRbcS* promoter sequence was searched against PLACE and PlantCARE databases, and the results are shown in Figure 1. The eukaryotic consensus TATA-box was identified at −28 nucleotides upstream of the transcription start site (TSS). The *AcRbcS* promoter contained several putative *cis*-acting elements, which included some representative light-responsive elements such as G-box [27–30] at position −675, Sp1 motif [31] at position −633, and six copies of SORLIP2 (sequences over-represented in light-induced promoter) [29] at position −706, −549, −512, −451, −297 and −239. Moreover, the *AcRbcS* promoter also contained putative GC-rich element (CCGCCC) at position −177 and −105. TATA box, the critical binding site, can be found in most eukaryotic gene promoters while other *cis*-acting elements frequently appeared in photosynthesis gene promoters of higher plants and microalgae; their specific roles were clearly described by a number of reports which concluded that these elements were essential for the light-responsive expression. It was interesting to note that G-box and Sp1 motif located in the upstream region, far from the transcription start site, but six copies of element SORLIP2 were found throughout the *AcRbcS* promoter region. The repetitive distribution of this element implied that *AcRbcS* promoter might be an active promoter. It was also noted that the GC-rich element was not found in the *RbcS* promoter of other green microalgae but it was found in *RbcS* promoter of *A. convolutus*, which suggested that the GC-rich element may not be essential for the light-responsive expression. Indeed, as described in a previous report, this element was found in maize *RbcS* promoter but not in rice *RbcS* promoter; the deletion of this element did not cause any strong effects on light-responsive expression of a reporter gene in maize mesophyll cells [32]. However, there was not enough proof to demonstrate the specific role of GC-rich elements in light-regulated gene promoters; therefore, the functional characterization of this element from light-regulated promoters should be carried out.

Sequence analysis of *AcRbcS* promoter also revealed the presence of other potential motifs such as the CAGAC-motif at position −467 and three copies of YCCYTGG-motifs at position −650, −605 and −457; both motifs were commonly found in the *RbcS* promoters of non-angiosperm plants. In addition, a potential CACCACA-motif which was apparent in the *RbcS* promoter of *Dunaliella tertiolecta* [5] was also recognized at position −668 bp of the *AcRbcS* promoter region. These findings suggested that these motifs were conserved and might play an important role in light-responsive gene expression in some of the green microalgae *RbcS* genes. However, deletion of these motifs in *RbcS* gene promoters of green microalgae species should be carried out to verify the specific functions of each motif.

Taken all together, the presence of several *cis*-acting elements known to be involved in photo-responsiveness of plant photosynthesis gene promoters and green microalgae *RbcS* gene in the upstream regions of the predicted transcription start site indicated that the isolated *AcRbcS* promoter reported in this study could be a full and active promoter.

### 2.2. Determination of the Transcription Start Site

**Figure 2:**
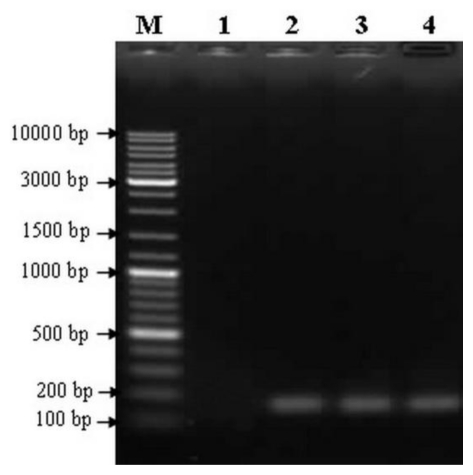
Determination of the transcription start site using 5′-RACE. Lane M: 2-log DNA marker (New England Biolabs, UK); Lane 1: negative control using total RNA without reverse transcriptase; Lanes 2−4: SMARTer RACE PCR products from different cDNA as templates.

The putative transcription start site (TSS, +1) of *AcRbcS* mRNA was determined using the SMARTer RACE PCR approach. A single band of 167 bp was obtained and cloned into pGEM-T Easy vector (Figure 2). Upon sequencing, this fragment exhibited complete homology to the 28 bases in the 5′ untranslated region of the previously cloned *AcRbcS* cDNA [26]. Nucleotide sequence analysis of three independent clones indicated that *AcRbcS* mRNA initiates 52 nucleotides upstream from the translation start codon (ATG). This result was also consistent with that predicted by using Promoter 2.0 and TSSP-TCM programs.

### 2.3. Detection of Transgenes Using PCR Analysis

**Figure 3:**
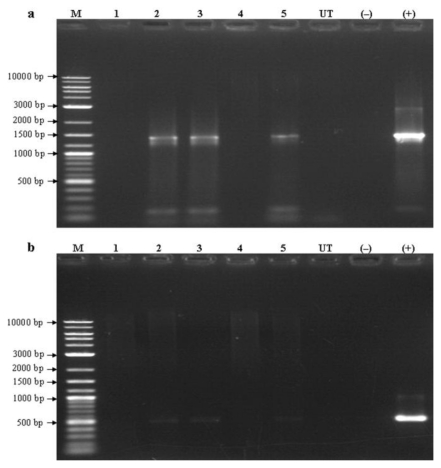
PCR analysis of putative transformed lines. (**a**) and (**b**) The presence of β-glucuronidase (*gusA*) and hygromycin phosphotransferase (*hpt*) genes were detected using *gus*- and *hpt*-specific primers, respectively. Three out of five putative lines tested (lines 2, 3 and 5) showed the presence of both *gusA* and *htp* genes. Lane M: 2-log DNA marker (New England Biolabs, UK); Lanes 1−5: putative transformed lines; Lane UT: untransformed control; Lane (−): negative control (without DNA template); Lane (+): positive control (pAcRbcS::gusA plasmid DNA).

Recently, many techniques have been developed to detect transgenes in transgenic organisms. Among these techniques, PCR-based techniques followed by gel electrophoresis and detection are routinely used to detect transgenes in plants as well as in microalgae [33,34]. Besides, other techniques such as the use of molecular beacon assay [35], fluorescence *in situ* hybridization [36] was also used. In the present study, the isolated *AcRbcS* promoter sequence was used to drive the expression of the transgenes in *A. convolutus*. The preliminary analysis of putative transformed *A. convolutus* lines was carried out using PCR. Two primer sets designed based on the coding sequence of β-glucuronidase (*gusA*) and hygromycin phosphotransferase (*hpt*) genes from destination vector pMDC163 were used in subsequent PCR analysis. Amplified with these transgene-specific primers, three out of five putative transformed lines (lines 2, 3 and 5) gave expected amplified products of 1433 bp for *gusA* (Figure 3a) and 509 bp for *hpt* gene (Figure 3b). This indicated the presence of *gusA* and *hpt* in the genome of these transformed lines. In contrast, no bands were detected in the untransformed cell and negative control (without DNA template) (Figure 3). The failure in detection of the transgenes in two putative transformed lines (Figure 3, lines 1 and 4) could have resulted from the rearrangement of pAcRbcS::gusA T-DNA or the transient expression of transgene in *A. convolutus*.

### 2.4. Integration of the Transgenes Driven by the *AcRbcS* Promoter

It is known that efficient promoters, including species-specific and universal promoters, are essential for foreign gene expression. For instance, the native ubiquitin promoter is more efficient than the heterologous CaMV35S promoter in driving the expression of foreign genes in monocot plant [37]. The regulatory region of the *RbcS* gene is an excellent candidate for the development of a promoter that can be widely used to achieve a high level of transgene expression in green algae as well as eukaryotic organisms [3,5,38,39].

**Figure 4:**
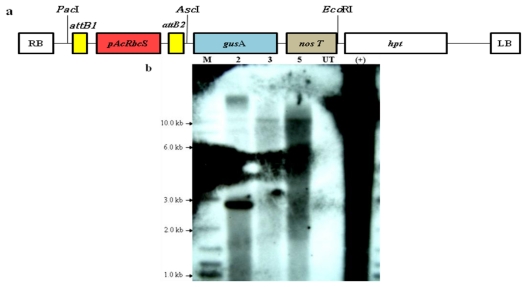
Southern hybridization analysis of transformed *A. convolutus*. (**a**) The presentation of the T-DNA region with *AcRbcS* promoter fused with *gusA* and *hpt* genes. The position of *Eco*RI restriction site was indicated; (**b**) Genomic DNA (15 μg) from three PCR-positive transformed lines (lines 2, 3 and 5) and untransformed cell was digested with *Eco*RI restriction enzyme and probed with a biotin-labeled *gusA* gene-specific probe. Lane M: 2-log DNA marker (New England Biolabs, UK); Lane UT: untransformed (negative control); Lane (+): pAcRbcS::gusA plasmid DNA (positive control, 10 μg linearized using *Eco*RI restriction enzyme).

In order to confirm the integration of the transgenes into the nuclear genome of *A. convolutus*, Southern hybridization analysis has been carried out. Approximately 15 μg genomic DNA isolated from the untransformed *A. convolutus* and three PCR-positive transformed lines was digested with *Eco*RI restriction enzyme and then hybridized with the *gusA*-gene-specific probe. *Eco*RI was chosen since it does not interfere with the *gusA* gene and has only one restriction site on the expression vector pAcRbcS::gusA (Figure 4a), which helped to linearize the expression vector when it was totally digested. In addition, after being digested with this enzyme, the genome of transgenic *A. convolutus* gave good fragmentation which was sufficient for Southern blot hybridization. Further, with the restriction site of *Eco*RI being located outside the *gus*A gene, this ensured that the copy number of the transgenes were determined precisely. The result showed that all of transformed lines yielded one or more bands, while no hybridization signal was detected from untransformed *A. convolutus* (Figure 4b). This was tangible evidence for the integration of transgenes into the nuclear genome of *A. convolutus*. The result also demonstrated that these transgenic lines may contain one to four copies in the nuclear genomes, which revealed that the transgenes could be integrated randomly into the genome, and *gus*A gene served as a strong selection for successful transformation of this alga. An identical hybridized banding pattern shown in two of the transformed lines (Figure 4, lines 3 and 5) suggested that these lines might be derived from a single transformed cell, which later divided into two cells during the overnight recovery step, before plated onto selection medium. This is the first report using the native promoter to drive the expression of the foreign genes in green microalga *A. convolutus*. The fact that the *AcRbcS* promoter functioned in *A. convolutus* suggested that this promoter could be used to establish a transformation system for *A. convolutus* to develop this microalga as an alternative host for the expression of heterologous proteins.

### 2.5. Influence of Light on the Regulation of *AcRbcS* Promoter Activity

It is known that the expression level of *RbcS* mRNA is regulated by light in plants [40] as well as green microalgae [26,41]. The fusion of *RbcS* promoters to reporter genes has been used to study light regulation in transgenic plants [38,39] but no such reports dealt with green algae.

**Figure 5:**
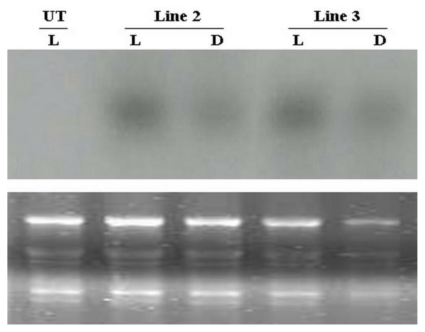
Light regulation of the *AcRbcS* promoter in transformed *A. convolutus*. Total RNA loaded on the gel was hybridized with a *gusA*-specific probe (**upper panel**). Total RNA was isolated from two transformed lines (lines 2 and 3) and an untransformed cell (UT) grown under continuous light for 3 days after 1-day subculture (L) and then transferred to dark for 24 h (D). Ethidium bromide staining of ribosomal RNA samples used to demonstrate equivalent loading (**lower panel**).

In order to support the confirmation of integration of transgenes as well as to determine whether the *AcRbcS* promoter showed light regulation in transgenic *A. convolutus*, the levels of *gus*A transcript were preliminary examined in *A. convolutus* grown in continuous light for 3 days after 1-day subculture (L) and then transferred to dark condition for 24 h (D). This light regime has previously been used to examine the expression level of *RbcS* mRNA in *A. convolutus* with minor modification in this study, where 24 h was used instead of 12 h in dark conditions. Under such a regime, the expression level of *A. convolutus RbcS* gene was highest in continuous light for 3 days after 1-day subculture (L) and then reduced after 12 h in the dark [26]. In this study, the expression of *gus*A gene at RNA level was examined in two out of three transformed lines. As can be seen from the results, *gus*A mRNA was clearly present in all transgenic *A. convolutus* lines before being placed in dark conditions (Figure 5). After 24 h in total darkness, however, *gus*A transcript levels in these transgenic lines were drastically decreased (Figure 5), suggesting a marked down-regulation of the *AcRbcS* promoter in the absence of light. It implied that the *gus*A gene has been integrated into the genome of transgenic *A. convolutus*. It also revealed that the activity of *AcRbcS* promoter obviously depended on light. This suggested that the *AcRbcS* promoter seems to be light-regulated in transgenic *A. convolutus* as demonstrated by the large decrease of corresponding *gus*A transcripts. This pattern was also reported in the other studies conducted on higher plants, of which the expression levels of *gus*A gene in leaves driven by the *RbcS* promoters also reduced after being transferred from the light to the dark [38,39].

## 3. Experimental Section

### 3.1. Culture Conditions

Green microalga *Ankistrodesmus convolutus* was collected from axenic freshwater and deposited in the Department of Cell and Molecular Biology, Faculty of Biotechnology and Biomolecular Sciences, Universiti Putra Malaysia. The culture is grown and maintained in Bold’s Basal Medium (BBM) [42]. Cultures were agitated on a gyratory shaker (150 rpm) at 25 °C without aeration under illumination at a light intensity of 55 μmol photons m^−2^ s^−1^ with a photocycle of 12 h light/12 h dark.

### 3.2. Isolation and Sequence Analysis of *AcRbcS* Promoter

The *AcRbcS* promoter region from *A. convolutus* was obtained by TAIL-PCR amplification and cloned into pGEM-T Easy vector (Promega, USA) as described in our previous report [43]. Plasmid DNA was isolated using PureYield Plasmid Midiprep System (Promega, USA) according to the manufacture’s instruction and sequenced commercially. The location and distribution of putative *cis*-acting elements were analyzed by using the Plant *Cis*-acting Regulatory DNA Elements (PLACE) [44] and Plant *Cis-*acting Regulatory Elements (PlantCARE) [45] databases.

### 3.3. Identification of the Transcription Start Site by 5′-Rapid Amplification of cDNA Ends (5′-RACE)

Total RNA was isolated from *A. convolutus* as described in our previous report [46]. The 5′ end of *AcRbcS* cDNA was determined using the SMARTer^™^ RACE cDNA Amplification Kit (Clontech, USA) according to the manufacturer’s instructions. Briefly, approximately 1 μg of total RNA was converted into first-strand cDNA using SMARTScribe RT at 42 °C for 1.5 h. The 5′-RACE-ready cDNA was obtained using the 5′-CDS Primer A and SMARTer IIA oligonucleotides supplied in the kit. Upon reaching the end of the mRNA template, the terminal transferase activity of SMARTScribe RT adds several dC residues to the 3′ end of the first-strand cDNA, and the SMARTer oligonucleotides contains a terminal stretch of modified bases that anneal to the extended cDNA tail, allowing the oligonucleotides to serve as a template for the reverse transcription. A touchdown PCR was performed with the Universal Primer A Mix (UPM) from the kit and the first gene-specific primer (RGSP1: 5′-CGCGATCTGCTCGTCGTTCA-3′) as the forward and reverse primers, respectively. The PCR program consisted of 5 cycles of 94 °C for 30 s and 72 °C for 3 min, followed by 5 cycles of 94 °C for 30 s, 70 °C for 30 s and 72 °C for 3 min, then ended with 25 cycles of 94 °C for 30 s, 68 °C for 30 s and 72 °C for 3 min. The nested PCR reaction was then conducted with the Nested Universal Primer A (NUP) provided in the kit and the second gene-specific primer (RGSP2: 5′-TTCACGGGCTGCCACACCAT-3′) as the forward and reverse primers, respectively. The amplification was performed through 30 cycles of 94 °C for 30 s, 62 °C for 45 s and 72 °C for 1 min, followed by a final elongation step of 72 °C for 5 min. After separation on 1.2% (w/v) agarose gel electrophoresis, the obtained single band of approximately 170 bp was excised from the gel, purified using QiaquickGel Extraction Kit (Qiagen, Germany), cloned into pGEM-T Easy vector (Promega, USA) and sequenced commercially. Identical nucleotide sequence corresponding to the 5′ end of *AcRbcS* cDNA was obtained by sequence analysis of three independent clones.

### 3.4. Construction of Promoter::gusA Fusing Vector

**Figure 6:**
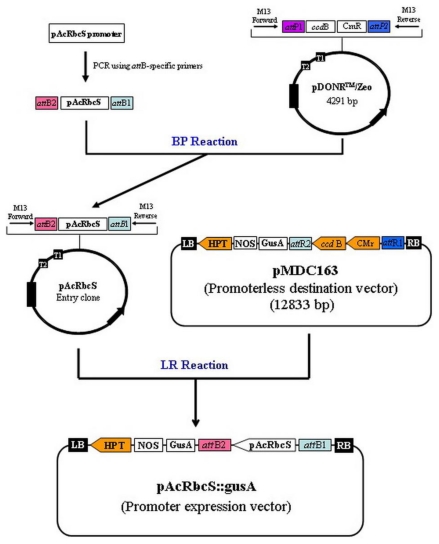
Schematic diagram showing the construction of pAcRbcS::gusA vector.

The construction of promoter::gusA fusing vector was made using the Gateway^®^ cloning technology with Clonase^™^ II (Invitrogen, USA) according to the manufacturer’s instructions. A schematic diagram for the construction of binary vector was depicted in Figure 6. PCR amplification of *AcRbcS* promoter was performed using a promoter-specific primer set: pAcRbcS-F (5′-GGGGAC AAGTTTGTACAAAAAAGCAGGCTGCACCACCGCAGCTTAGCGCCCA-3′) and pAcRbcS-R (5′-G GGGACCACTTTGTACAAGAAAGCTGGGTCATTGCTGCTGCTTGCGGGTGA-3′), in which the *att*B1 and *att*B2 sites (underlined) were added to the 5′-ends of the forward and reverse primers, respectively. The PCR reaction consists of 1× *Pfu* buffer, 0.2 mM dNTPs mix, 0.2 μM forward primer, 0.2 μM reverse primer, 0.5 U *Pfu* DNA polymerase (Fermentas, USA) and 100 ng plasmid DNA. PCR conditions were initial denaturing at 95 °C for 5 min, followed by 35 cycles of 95 °C for 45 s, 68 °C for 45 s and 72 °C for 1 min, and a final elongation step of 72 °C for 7 min. The obtained PCR fragment was recombined into the entry vector pDONR/Zeo using a BP clonase enzyme mixture. The BP recombination reaction was then transformed into *E. coli* DH5α competent cells by electroporation and zeocin-resistant colonies were selected. Plasmid DNA was isolated and used for a second reaction with the LR clonase enzyme mixture, in this reaction the promoter fragment was recombined into the promoterless destination vector pMDC163 containing the β-glucuronidase (*gusA*) reporter gene to create the promoter expression vector, pAcRbcS::gusA. *E. coli* DH5α was transformed with pAcRbcS::gusA, the resulted recombinant plasmid was verified by sequencing and introduced into *Ankistrodesmus convolutus* using electroporation method.

### 3.5. Nuclear Transformation of *A. convolutus*

Cells in early stationary phase were collected by centrifugation at 6000 *g* for 5 min, treated with cellulase (2%) and pectinase (0.3%) to partially remove cell wall. Enzymes treated cells were electroporated using the Electroporator 2510 (Eppendorf, Germany) according to the method described by Chi *et al.* (unpublished). Briefly, the enzymes treated cells were resuspended in electroporation buffer (5 mM CaCl_2_, 20 mM HEPES, 200 mM sorbitol and 200 mM mannitol, pH 7.0) to the final cell density of 1–2 × 10^6^ cells/mL. A total of 2 μg pAcRbcS::gusA and 6 μg carrier DNA (salmon sperm DNA) were added to cell suspension. The resuspended cells were transferred into electroporation chamber and electroporated at a pulse voltage of 1.8 kV with the constant pulse duration of 5 ms using the Electroporator 2510 (Eppendorf, Germany). After electroporation, the cells were transferred into 5 mL of liquid BBM medium for recovery for 24 h in the dark and later they were spread onto solid BBM plates supplemented with hygromycin (40 μg/mL), and grown at 25 °C under 12:12 h light-dark cycle.

Transformed colonies appeared in a week, and independent colonies were maintained on selection medium. The hygromycin resistant colonies were then cultured in liquid BBM supplementing hygromycin (40 μg/mL) at 25 °C with agitation on a gyratory shaker (150 rpm) without aeration under 55–60 μmol photons m^−2^ s^−1^ illumination with a photocycle of 12 h light/12 h dark.

### 3.6. Analysis of T-DNA Integration and Transgenes Expression

Total RNA and genomic DNA of transgenic A. convolutus were extracted as described in our previous reports [46,47]. Putative transformed lines were initially screened by PCR, which was performed with two set of primers: gus-F (5′-ACCGAAGTTCATGCCAGTCCAGCG-3′) and gus-R (5′-ATGTCACGCCGTATGTTATTGCCG-3′) specific to β-glucuronidase (*gusA*) gene to amplify a 1433-bp fragment, and hpt-F (5′-AGCTGCGCCGATGGTTTCTACAA-3′) and hpt-R (5′-ATCGCCT CGCTCCAGTCAATG-3′) to amplify a 509-bp fragment of the hygromycin phosphotransferase (*hpt*) gene.

The transformed lines were also analyzed by Southern hybridization. Approximately 15 μg of genomic DNA was digested with *Eco*RI overnight, electrophoresed on 0.8% (w/v) agarose gel. The DNA was then overnight transferred by capillarity onto nylon membrane (Hybond N^+^, Amersham Bioscience) with 0.4 N NaOH, and autocrosslinked by UV-crosslinker (Ultra-Violet Products, UK) at the wavelength of 254 nm for 2 min. The membrane with cross-linked DNA was prehybridized at 50 °C for 2 h before hybridized at 55 °C overnight using the biotin-labelled *gusA* gene fragment as probe. This probe was prepared by PCR amplification of the *gusA* gene using gus-F and gus-R primers and incorporating biotin-14-dCTP. The hybridized membrane was washed twice with low stringency washing solution (2× SSC, 0.1% (w/v) SDS) for 15 min, and twice with medium stringency washing solution (0.5× SSC, 0.1% (w/v) SDS) for 30 min at 65 °C. The membrane was exposed to X-ray film (Kodak Medical X-ray Film, Kodak) in an X-ray cassette before developing the film for signal visualization.

For Northern hybridization analysis, approximately 15 μg of total RNA from selected transformed lines was denatured and separated in 1.2% (w/v) agarose gel containing 6% (v/v) formaldehyde followed by staining with ethidium bromide. The RNA samples was then transferred overnight by capillarity with 10× SSC buffer onto nylon membrane (Hybond N^+^, Amersham Bioscience) and autocrosslinked by UV-crosslinker (Ultra-Violet Products, UK) at the wavelength of 254 nm for 2 min. The blotted membrane was hybridized at 67 °C overnight with the labeled probe for *gusA* gene prepared by PCR incorporation of biotin-14-dCTP. The hybridized membrane was washed twice with 2× SSC, 0.1% (w/v) SDS for 15 min, and twice with 0.5× SSC, 0.1% (w/v) SDS for 30 min at 67 °C. The membrane was exposed to X-ray film (Kodak Medical X-ray Film, Kodak) in an X-ray cassette before developing the film for signal visualization.

## 4. Conclusions

Green algae have proven their utility and tractability as a system for the production of therapeutic or industrial proteins and peptides. They now seem poised to become the “green” alternative to the current mammalian, yeast, or bacterial recombinant protein production systems. Development of an expression system using green microalga *Ankistrodesmus convolutus* is worthwhile because of its importance as a potential model organism for genetic engineering. Like other unicellular green algae, *A. convolutus* meets the characteristics required for production of recombinant proteins, its cells grow fast in a simple medium and this species can be cultured simply, rapidly and economically.

In the present study, the *AcRbcS* promoter sequence which was isolated from green microalga *A. convolutus* in our previous work has been analyzed and then used to drive the expression of *gusA* in this microalga species. The presence of several *cis*-acting elements and motifs known to be involved in the light-dependent expression of photosynthesis genes of higher plants and other green microalgae species, and the retrieval of the transformed *A. convolutus* expressing transgenes driven by *AcRbcS* promoter suggested that the isolated promoter has contained the promoter elements necessary for the transcription and regulation of gene expression. The transformation construct may be functional in other closely related species. Indeed, describing the use of promoter-constructs from a closely related species to develop a nuclear transformation has been recently reported [5]; it is likely that the *AcRbcS* promoter and the construct developed in this study may be useful as a universal promoter and in the development of a transformation system, respectively, for other green microalgae species such as *Chlamydomonas reinhardtii* and *Dunaliella* spp.

In this study, it was found that the *AcRbcS* promoter is regulated by light as demonstrated by a reduction of corresponding *gusA*-specific transcripts when the transgenic *A. convolutus* was transferred from light to dark conditions; however, further experiments, such as determination of the presence of any negative regulatory elements within the *AcRbcS* promoter sequence, should be performed to increase the expression levels of the transgenes under the control of this promoter. This work demonstrates, for the first time, the use of the native promoter to drive the expression of foreign genes in nuclear genome of green microalga *A. convolutus*. The achievement of a transformation system and the practical application of the *AcRbcS* promoter have reliably contributed to the development of *A. convolutus* as a model organism for algae research, and it is possible to develop this microalga as an expression system for the production of recombinant proteins.

## References

[1] Mayfield S.P., Franklin S.E., Lerner R.A. (2003). Expression and assembly of a fully active antibody in algae. Proc. Natl. Acad. Sci. USA.

[2] Griesbeck C., Kobl I., Heitzer M. (2006). *Chlamydomonas reinhardtii*: A protein expression system for pharmaceutical and biotechnological proteins. Mol. Biotechnol.

[3] Stevens D.R., Rochaix J.D., Purton S. (1996). The bacterial phleomycin resistance gene *ble* as a dominant selectable marker in *Chlamydomonas*. Mol. Gen. Genet.

[4] Schroda M., Blocker D., Beck C.F. (2000). The *HSP70A* promoter as a tool for the improved expression of transgenes in *Chlamydomonas*. Plant J.

[5] Walker T.L., Becker D.K., Collet C. (2005). Characterisation of the *Dunaliella tertiolecta RbcS* genes and their promoter activity in *Chlamydomonas reinhardtii*. Plant Cell Rep.

[6] Lu Y., Li J., Xue L., Yan H., Yuan H., Wang C (2010). A duplicated carbonic anhydrase 1 (*DCA1*) promoter mediates the nitrate reductase gene switch of *Dunaliella salina*. J. Appl. Phycol.

[7] Tang D.K.H., Qiao S.Y., Wu M. (1995). Insertion mutagenesis of *Chlamydomonas reinhardtii* by electroporation and heterologous DNA. Biochem. Mol. Biol. Int.

[8] El-Sheekh M.M. (1999). Stable transformation of the intact cells of *Chlorella kessleri* with high velocity microprojectiles. Biol. Plant.

[9] Kim D.H., Kim Y.T., Cho J.J., Bae J.H., Hur S.B., Hwang I., Choi T.J. (2002). Stable integration and functional expression of flounder growth hormone gene in transformed microalga, *Chlorella ellipsoidea*. Mar. Biotechnol.

[10] Tan C., Qin S., Zhang Q., Jiang P., Zhao F. (2005). Establishment of a microparticle bombardment transformation system for *Dunaliella salina*. J. Microbiol.

[11] Feng S., Xue L., Liu H., Lu P. (2009). Improvement of efficiency of genetic transformation for *Dunaliella salina* by glass beads method. Mol. Biol. Rep.

[12] Blankenship E., Kindle K. (1992). Expression of chimeric genes by the light-regulated *cabII-1* promoter in *Chlamydomonas reinhardtii*: A *cabII-1/nit1* gene functions as a dominant selectable marker in a nit1 nit2 strain. Mol. Cell. Biol.

[13] Butanaev A.M. (1994). Hygromicin phosphotransferase gene as a dominant selective marker for transformation of *Chlamydomonas reinhardtii*. Mol. Biol.

[14] Teng C., Qin S., Liu J., Yu D., Liang C., Tseng C. (2002). Transient expression of *lacZ* in bombarded unicellular green alga *Haematococcus pluvialis*. J. Appl. Phycol.

[15] Chen Y., Wang Y., Sun Y., Zhang L., Li W. (2001). Highly efficient expression of rabbit neutrophil peptide-1 gene in *Chlorella ellipsoidea* cells. Curr. Genet.

[16] Franklin S., Ngo B., Efuet E., Mayfield S.P. (2002). Development of a *GFP* reporter gene for *Chlamydomonas reinhardtii* chloroplast. Plant J.

[17] Shao N., Bock R. (2008). A codon-optimized luciferase from *Gaussia princeps* facilitates the *in vivo* monitoring of gene expression in the model alga *Chlamydomonas reinhardtii*. Curr. Genet.

[18] Fischer N., Rochaix J.D. (2001). The flanking regions of PsaD drive efficient gene expression in the nucleus of the green alga *Chlamydomonas reinhardtii*. Mol. Genet. Genomics.

[19] Kindle K.L. (1990). High-frequency nuclear transformation of *Chlamydomonas reinhardtii*. Proc. Natl. Acad. Sci. USA.

[20] Cerutti H., Johnson A.M., Gillham N.W., Boynton J.E. (1997). A eubacterial gene conferring spectinomycin resistance on *Chlamydomonas reinhardtii*: integration into the nuclear genome and gene expression. Genetics.

[21] Auchincloss A.H., Loroch A.I., Rochaix J.D. (1999). The argininosuccinate lyase gene of *Chlamydomonas reinhardtii*: cloning of the cDNA and its characterization as a selectable shuttle marker. Mol. Gen. Genet.

[22] Fuhrmann M., Oertel W., Hegemann P. (1999). A synthetic gene coding for the green fluorescent protein (*GFP*) is a versatile reporter in *Chlamydomonas reinhardtii*. Plant J.

[23] Kovar J.L., Zhang J., Funke R.P., Weeks D.P. (2002). Molecular analysis of the acetolactate synthase gene of *Chlamydomonas reinhardtii* and development of a genetically engineered gene as a dominant selectable marker for genetic transformation. Plant J.

[24] Sun Y., Yang Z.Y., Gao X.S., Li Q.Y., Zhang Q.Q., Xu Z.K. (2005). Expression of foreign genes in *Dunaliella* by electroporation. Mol. Biotechnol.

[25] Chu W.L., Phang S.M., Goh S.H., Blakebrough N, Shaari K., Kadir A.A., Ali A.R.M. Promising Microalgae for Production of Useful Chemicals. Proceeding of the Conference on Medicinal Products from Tropical Rain Forests.

[26] Thanh T., Chi V.T.Q., Abdullah M.P., Omar H., Noroozi M., Ky H., Napis S. (2011). Construction of cDNA library and preliminary analysis of expressed sequence tags from green microalga *Ankistrodesmus convolutus* Corda. Mol. Biol. Rep.

[27] Giuliano G., Pechersky E., Malik V.S., Timko M.P., Scolnik P.A., Cashmore A.R. (1988). An evolutionarily conserved protein binding sequence upstream of a plant light-regulated gene. Proc. Natl. Acad. Sci. USA.

[28] Williams M.E., Foster R., Chua N.H. (1992). Sequences flanking the hexameric G-box core CACGTG affect the specificity of protein binding. Plant Cell.

[29] Hudson M.E., Quail P.H. (2003). Identification of promoter motifs involved in the network of phytochrome A-regulated gene expression by combined analysis of genomic sequence and microarray data. Plant Physiol.

[30] Li T., Gong C., Wang T. (2010). The rice light-regulated gene *RA68* encodes a novel protein interacting with oxygen-evolving complex PsbO mature protein. Plant Mol. Biol. Rep.

[31] Ahrazem O., Rubio-Moraga A., López R.C., Gómez-Gómez L. (2010). The expression of a chromoplast-specific lycopene beta cyclase gene is involved in the high production of saffron’s apocarotenoid precursors. J. Exp. Bot.

[32] Nomura M., Katayama K., Nishimura A., Ishida Y., Ohta S., Komari T., Miyao-Tokutomi M., Tajima S., Matsuoka M. (2000). The promoter of *rbcS* in a C3 plant (rice) directs organ-specific, light-dependent expression in a C4 plant (maize), but does not confer bundle sheath cell-specific expression. Plant Mol. Biol.

[33] Higuchi R., Dollinger G., Walsh P.S., Griffith R. (1992). Simultaneous amplification and detection of specific DNA sequences. Nat. Biotechnol.

[34] Kumar S.C., Misqitta R.W., Reddy V.S., Rao B.J., Rajam M.V. (2004). Genetic transformation of the green alga *Chlamydomonas reinhardtii* by *Agrobacterium tumefaciens*. Plant Sci.

[35] Kota R., Holton T.A., Henry R.J. (1999). Detection of transgenes in crop plants using molecular beacon assays. Plant Mol. Biol. Rep.

[36] Jin W.W., Li Z.Y., Fang Q., Altosaar I., Liu L.H., Song Y.C. (2002). Fluorescence *in situ* hybridization analysis of alien genes in *Agrobacterium*-mediated *Cry1A(b)*-transformed rice. Ann. Bot.

[37] Wang J., Oard J.H. (2003). Rice ubiquitin promoters: Deletion analysis and potential usefulness in plant transformation systems. Plant Cell Rep.

[38] Gittins J.R., Pellny T.K., Hiles E.R., Rosa C., Biricolti S., James D.J. (2000). Transgene expression driven by heterologous ribulose-1,5-bisphosphate carboxylase/oxygenase small-subunit gene promoters in the vegetative tissues of apple (*Malus pumila* Mill.). Planta.

[39] Marraccini P., Courjault C., Caillet V., Lausanne F., Lepage B., Rogers W.J., Tessereau S., Deshayes A. (2003). Rubisco small subunit of *Coffea arabica*: cDNA sequence, gene cloning and promoter analysis in transgenic tobacco plants. Plant Physiol. Biochem.

[40] Terzaghi W.B., Cashmore A.R. (1995). Light-regulated transcription. Annu. Rev. Plant Physiol. Plant Mol. Biol.

[41] Yamazaki T., Yamamoto M., Sakamoto W., Kawano S. (2005). Isolation and molecular characterization of *rbcS* in the unicellular green alga *Nannochloris bacillaris* (Chlorophyta, Trebouxiophyceae). Phycol. Res.

[42] Nichols H.W., Stein J.R. (1973). Growth Media-Freshwater. Handbook of Phycological Methods: Culture Methods and Growth Measurements.

[43] Thanh T., Chi V.T.Q., Abdullah M.P., Omar H., Napis S (2011). Efficiency of the ligation-mediated PCR and TAIL-PCR methods on isolation of *RbcS* promoter sequences from green microalga *Ankistrodesmus convolutus*. Mol. Biol.

[44] Higo K., Ugawa Y., Iwamoto M., Korenaga T. (1999). Plant *cis*-acting regulatory DNA elements (PLACE) database. Nucleic Acids Res.

[45] Lescot M., Déhais P., Thijs G., Marchal K., Moreau Y., van de Peer Y., Rouzé P., Rombauts P. (2002). PlantCARE, a database of plant *cis*-acting regulatory elements and a portal to tools for *in silico* analysis of promoter sequences. Nucleic Acids Res.

[46] Thanh T., Omar H., Abdullah M.P., Chi V.T.Q., Noroozi M., Ky H., Napis S. (2009). Rapid and effective method of RNA isolation from green microalga *Ankistrodesmus convolutus*. Mol. Biotechnol.

[47] Thanh T., Chi V.T.Q., Abdullah M.P., Omar H., Noroozi M., Napis S (2011). Cloning and characterization of ribulose-1,5-bisphosphate carboxylase/oxygenase small subunit (*RbcS*) cDNA from green microalga *Ankistrodesmus convolutus*. Mol. Biol. Rep.

